# Complete esophageal obstruction following endoscopic variceal band ligation

**DOI:** 10.3402/jchimp.v3i1.20043

**Published:** 2013-04-17

**Authors:** Harjit Chahal, Anita Ahmed, Carlton Sexton, Abhijit Bhatia

**Affiliations:** 1 Department of Medicine, Medstar Union Memorial Hospital, Baltimore, MD, USA; 2 Department of Radiology, Medstar Union Memorial Hospital, Baltimore, MD, USA; 3 Department of Gastroenterology, Medstar Union Memorial Hospital, Baltimore, MD, USA

**Keywords:** esophageal obstruction, band ligation, endoscopy

## Abstract

Variceal hemorrhage is a potential complication of portal hypertension. Besides medical management, endoscopic variceal band ligation (EVBL) has emerged as a promising prophylactic tool proving to be superior to sclerotherapy. EBVL is a simple procedure associated with minor complications and short recovery time. In this report, we present a case of a rare complication of complete esophageal obstruction following an EVBL procedure. Given the high numbers of such procedures performed, it is imperative that internists and specialists be aware of this unusual complication.

Variceal hemorrhage is an ominous complication of portal hypertension. Annual incidence of upper GI bleed resulting from variceal hemorrhage is reported close to 5–10% in patients with cirrhosis (1). Small varices gradually increase in size and develop into larger varices at a rate of up to 30% per year. Larger varices are associated with a higher risk of bleeding. Furthermore, while the risk of first bleeding is approximately 20%, a substantially higher risk of rebleed approaching close to 70% exists in such patients (2). Therefore, secondary prophylaxis of such events is essential in patients with existing varices.

Current therapies for the prevention of variceal hemorrhage include non-selective β-blocker therapy and endoscopic variceal ligation. In the past, variceal bleeding was managed by sclerotherapy that was associated with significant complications (3). Randomized controlled trials have shown that endoscopic variceal band ligation (EVBL) has fewer complications and improved survival vs. sclerotherapy (4). EVBL is a relatively simple procedure with minor complications and a short recovery time. Complications following EVBL are rare and may include post-procedure pain, esophageal dysfunction, local ulcers and stricture formation.

In this report, we present a case of an unusual complication following an EVBL procedure.

## Case presentation

### Patient presentation

A 54-year-old female with a medical history of hypertension, hepatitis C, and cirrhosis presented with nausea, vomiting and dysphagia of 1-day duration following surveillance esophagogastroduodenoscopy (EGD) and banding from an outside hospital. She reported that her symptoms started immediately post-procedure and that she continued to have multiple episodes of mostly clear vomit with streaks of blood. She also reported throat, chest, and epigastric pain and was unable to eat anything since the procedure due to her symptoms.

Her past medical history included hypertension, hepatitis C, cirrhosis, gastroesophageal reflux disease, variceal hemorrhage, gall stones and Graves disease. She was on propranolol, lactulose, alprazolam and amlodipine at home. Social history was significant for heavy alcohol use for over 30 years, which she quit following an episode of variceal bleeding. She had no episodes of recurrent gastrointestinal bleeding since her last EGD and banding. On physical examination, she was an ill-appearing female, had dry oral mucosa, tachycardic, had epigastric and RUQ tenderness with negative Murphy's sign, and no rebound or guarding. During the interview, she vomited small amounts of non-bloody white material several times.

On presentation, her laboratory tests were as follows: WBC 13.1, Hb/Hct 14/39.7, platelets 260; Na 152, K 3.6, Cl 112, CO_2_ 22, BUN 30, Cr 0.87, glc 146; lipase 93, Alk Phos 113, AST/ALT 48/75, lactic acid 2.0 and drug screen was positive for propofol.

### Hospital course

**Fig. 1:**
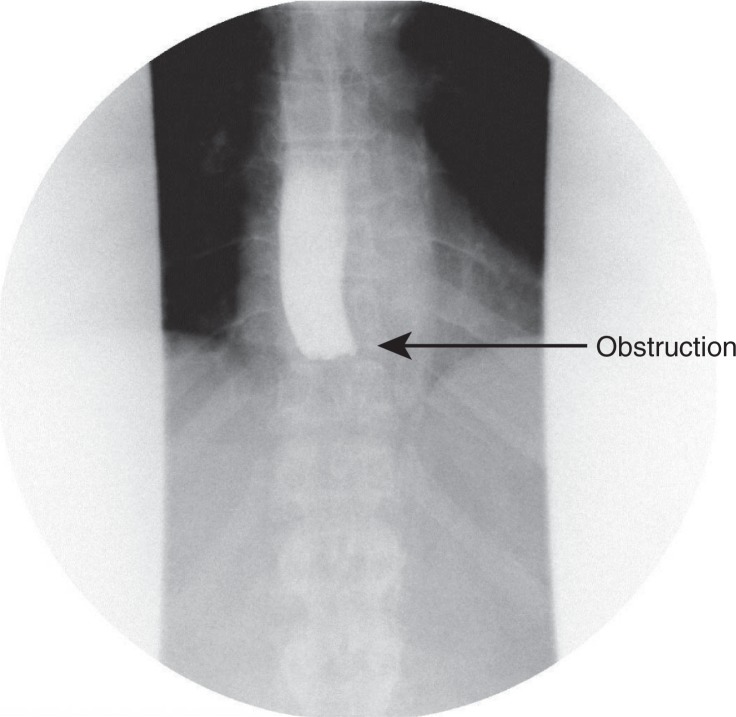
Barium swallow showing complete esophageal obstruction in the distal esophagus.

**Fig. 2:**
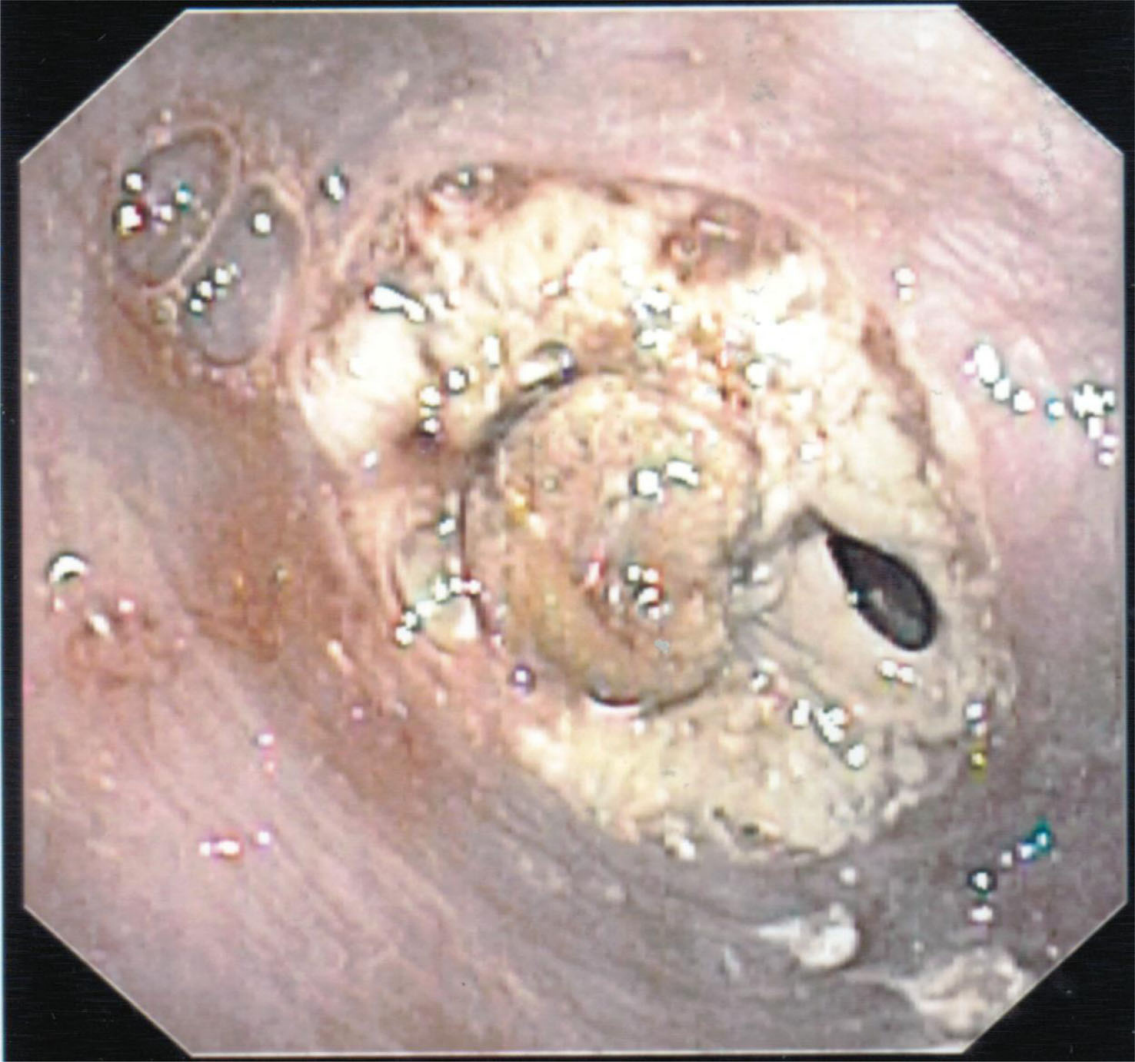
EGD showing complete obstruction with entrapped band and surrounding necrosis.

The patient was admitted for dehydration due to nausea and vomiting. She was started on IV fluids, anti-emetics, pain control and kept NPO (nothing by mouth). Her dyselectrolytemia improved following hydration and her diet was advanced on the following day. She could not tolerate oral intake and reported worsening dysphagia, nausea and vomiting. Over the course of next 2 days, the patient continued to have worsening dysphagia with no relief from anti-emetics. A barium swallow was administered which showed a complete obstruction of the distal esophagus (Fig. 1). The differential for obstruction was thought to be either from local edema from the banding procedure, impacted food or a hematoma from the procedure. An EGD was performed; the scope was advanced to 34 cm to the level of obstruction and it was noted to have a band with entrapped varix in the center of the lumen surrounded by fibrotic material closing off the rest of the lumen (Fig. 2). Several attempts were made with biopsy forceps to reopen the lumen that resulted in a small amount of bleeding and the scope was removed at this point. Conservative management of the patient continued, i.e., NPO with intravenous hydration and pain control. Her symptoms improved over the course of the next 3–4 days and she started tolerating diet advancement and was subsequently discharged.

**Fig. 3:**
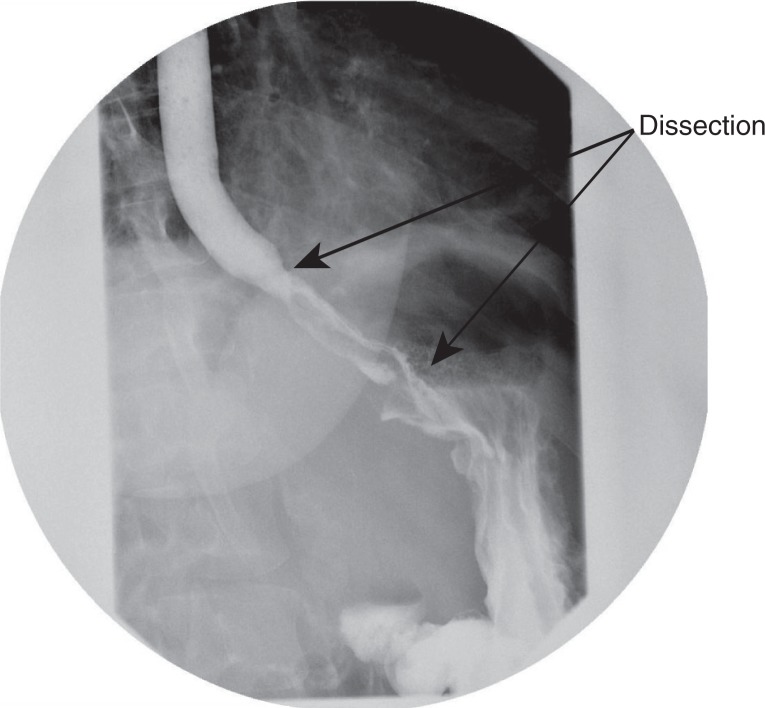
Repeat barium swallow showing esophageal intramural dissection and a parallel channel formation with no evidence of perforation.

She presented the next day with a recurrence of similar symptoms, i.e., dysphagia, abdominal pain and vomiting. A repeat barium swallow showed patency of the esophagus with a parallel channel that retained contrast much later than the initial swallow, suggestive of an esophageal tear with an intramural dissection of 6 cm to the level of gastro-esophageal junction (Fig. 3).

The patient was kept NPO with peripheral parenteral nutrition and close monitoring for possible esopheageal perforation. Over the course of the next several days, the diet was carefully advanced with inputs from gastroenterologist and surgical consultants. After 5 days, the patient reported much improvement in her symptoms and was able to tolerate a full diet for the first time.

**Fig. 4:**
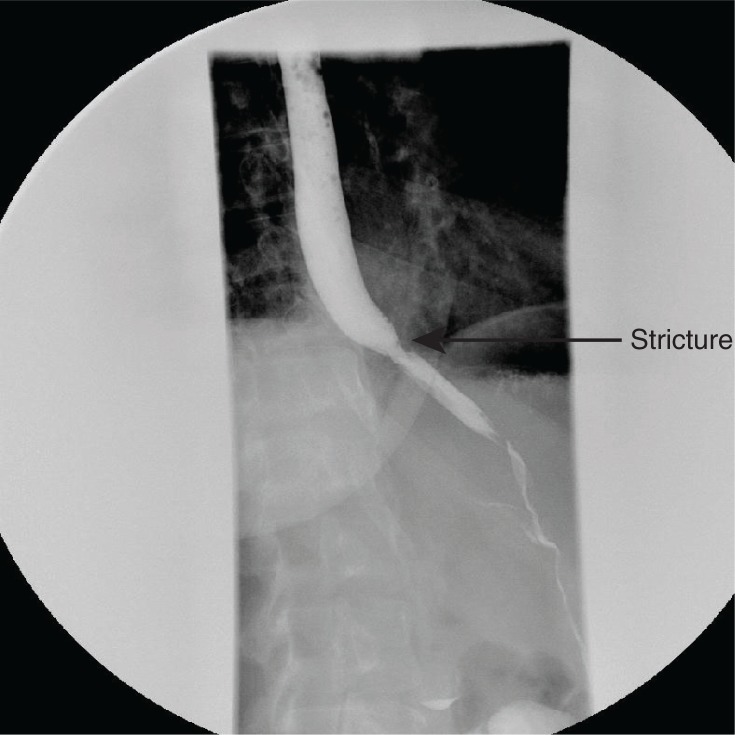
Barium swallow prior to discharge showing interval healing of dissection and a stricture at the site proximal to prior dissection.

A repeat barium swallow was obtained prior to discharge that showed no obstruction or dissection. A small stricture was noted at the proximal margin at the origin of the prior dissection (Fig. 4). She was discharged with subsequent follow-up appointment at the GI clinic.

## Discussion

In this report, we present a rarely reported complication from EVBL. So far, only three cases of this rare but dramatic complication have been reported to our knowledge and all three cases responded to conservative management with spontaneous resolution of the obstruction (5–7). Possible mechanisms for obstruction suggested in prior reports include local tissue edema and necrosis at banding site. Alternatively, a previously unrelated esophageal stricture such as a Schatzki ring could be obstructed by the peristaltic effect of esophagus pushing a proximal banded varix into it resulting in a ball-valve mechanism.

Unlike prior case reports, our case was the first case to have a complication following repeat EGD. In this case, we observed esophageal dissection and subsequent stricture formation. However, it is unclear as to whether the stricture was present before the EVBL or if it developed during healing from the dissection. Possible mechanisms for this complication could be premature resumption of diet and/or EGD manipulation of the area. Prior reports including the present case all showed improvement and complete recovery following conservative management. Based on current clinical reports, it could be concluded that this is a self-limiting complication. More information is needed to ascertain the factors that could predispose patients to this complication.

EBVL has essentially replaced sclerotherapy in the management of esophageal varices and studies have reported infrequent complications that are usually self-limiting. However, the number of EBVL procedures being performed has increased considerably and clinicians should be aware of more rare complications such as those described in this report.
